# Postoperative fluid retention after heart surgery is accompanied by a strongly positive sodium balance and a negative potassium balance

**DOI:** 10.14814/phy2.12807

**Published:** 2016-05-24

**Authors:** Lara Hessels, Annemieke Oude Lansink, Maurits H. Renes, Iwan C. C. van der Horst, Miriam Hoekstra, Daan J. Touw, Maarten W. Nijsten

**Affiliations:** ^1^ Department of Critical CareUniversity of GroningenUniversity Medical Center GroningenGroningenThe Netherlands; ^2^ Department of AnesthesiologyUniversity of GroningenUniversity Medical Center GroningenGroningenThe Netherlands; ^3^ Department of Clinical Pharmacy and PharmacologyUniversity of GroningenUniversity Medical Center GroningenGroningenThe Netherlands

**Keywords:** Extracellular volume, intracellular volume, osmolytes, potassium, sodium

## Abstract

The conventional model on the distribution of electrolyte infusions states that water will distribute proportionally over both the intracellular (ICV) and extracellular (ECV) volumes, while potassium homes to the ICV and sodium to the ECV. Therefore, total body potassium is the most accurate measure of ICV and thus potassium balances can be used to quantify changes in ICV. In cardiothoracic patients admitted to the ICU we performed complementary balance studies to measure changes in ICV and ECV. In 39 patients, fluid, sodium, potassium, and electrolyte‐free water (EFW) balances were determined to detect changes in ICV and ECV. Cumulatively over 4 days, these patients received a mean ± SE infusion of 14.0 ± 0.6 L containing 1465 ± 79 mmol sodium, 196 ± 11 mmol potassium and 2.1 ± 0.1 L EFW. This resulted in strongly positive fluid (4.0 ± 0.6 L) and sodium (814 ± 75 mmol) balances but in negative potassium (−101 ± 14 mmol) and EFW (−1.1 ± 0.2 L) balances. We subsequently compared potassium balances (528 patients) and fluid balances (117 patients) between patients who were assigned to either a 4.0 or 4.5 mmol/L blood potassium target. Although fluid balances were similar in both groups, the additionally administered potassium (76 ± 23 mmol) in the higher target group was fully excreted by the kidneys (70 ± 23 mmol). These findings indicate that even in the context of rapid and profound volume expansion neither water nor potassium moves into the ICV.

## Introduction

In clinical medicine, the conventional model on water and electrolyte distribution states that infused electrolyte‐free water (EFW) distributes proportionally over both the intracellular volume (ICV) and the extracellular volume (ECV) (Rose 2001; Guyton 2011; Frost 2015). The major cations of the ICV and ECV are potassium and sodium, respectively. Critically ill patients receive large electrolyte infusion volumes during treatment in the intensive care unit (ICU). Although retention of sodium and water are well‐known to accompany early ICU‐treatment (Moore 1959; Gosling 2003; Lindner et al. 2009; Silversides et al. 2010), the effect on potassium balance and ICV has not been studied. Since total body potassium (TBK) is considered as the gold standard for determining ICV (Patrick 1977; Finn et al. 1996; Monk et al. 1996; Dittmar and Reber 2004; Savalle et al. 2012), potassium balances could serve as a quantitative indicator of changes in TBK and thus ICV. We therefore performed fluid, sodium, potassium, and EFW balance studies in ICU patients to quantify changes in ICV and ECV. In addition, we also examined the effect of two different potassium supplementation protocols aiming for either a normal‐high or normal‐low potassium target on the potassium and fluid balances (Hoekstra et al. 2010, 2016).

## Methods

### Study design

In this study, we determined fluid, sodium, potassium, chloride, and EFW balances in critically ill patients admitted after cardiac surgery. The observational retrospective balance studies all involved patients of ≥18 years admitted to a tertiary cardiothoracic ICU from October 2010 until December 2014. Fluid, sodium, potassium, and chloride balances were derived from meticulously recorded input and output, including 24‐h urine collections. In all patients, potassium was regulated by our computerized potassium regulation protocol (Glucose and potassium Regulation in Intensive care Patients [GRIP‐II]) (Hoekstra et al. 2010). Patients were targeted to a serum potassium target of either 4.0 mmol/L (4.0 mmol/L target group) or 4.5 mmol/L (4.5 mmol/L target group) using our GRIP‐II protocol. Patients were assigned in alternating blocks during the GRIP‐COMPASS (computer‐driven Glucose and potassium Regulation program in Intensive care Patients with COMparison of PotASSium targets within normokalemic range) trial in substudy C (Hoekstra et al. 2010). Directly after completion of this trial our standard target was initially set at 4.5 mmol/L. However, after evaluation of the trial results it was subsequently set at 4.0 mmol/L, since the higher target conferred no clinical benefits (Hoekstra et al. 2016).

Patients who received renal replacement therapy were excluded from analysis. Our ICU did not have a full electronic patient database management system during the study period. Therefore, all data were derived from reviewing medical and nursing charts. Patients with missing or incomplete charts were excluded. Also, the required 24 h urine analysis was introduced at our ICU during the study period. Thus, we examined the various aspects of balances in complementary substudies A, B, and C, which enabled us to gather all information needed in as many patients as possible.

*Substudy A* evaluated patients in depth to establish the overall extent of cumulative fluid, sodium, potassium, chloride, and EFW retention during the first days after ICU admission. These variables were derived with comprehensive equations including all intake (IV fluids, nutrition, and medication) and excretion or losses (diuresis, insensible perspiration, drained fluids, and gastric retention [Tables 1, 2, 3]). Arterial pH and glucose level were also recorded, since marked changes in these parameters could affect potassium redistribution (Aronson and Giebisch 2011; Palmer 2015).

**Table 1:** Constants and calculations used in substudy A, B, and C

**Substudy A**
*Intake of water, sodium, chloride, and potassium*
Intake = infusion fluids + given medication + water (oral)
For electrolytes (mmol): volume × [electrolyte]_administered fluid_ (see Tables 2 and 3)
*Output of water, sodium, chloride, and potassium*
Output = gastric retention + drain production + insensible perspiration + diuresis (24 h urines)
For electolytes (mmol): volume × [electrolyte]_administered fluid_ (see Tables 2 and 3)
*Balance of water, sodium, chloride, and potassium*
Balance = intake − output
Gastric retention: volume × [electrolyte]_enteral/parenteral feeding_ (see Table 2)
Drain fluid loss: volume × mean blood [electrolyte]
Insensible perspiration: 10 mL/kg/day + 2.5 mL/kg/day per degree centigrade above 37°C (max body weight in equation: 100 kg) × 0.6 if intubated × 0.5 on admission day
Temperature: mean body temperature of the day (mean of temperature at 6 and 18 h)
*Blood (mmol/L)*
Blood potassium reference range 3.5–5.0
Mean blood potassium: 4.2
Blood sodium reference range 135–144
Mean blood chloride: 108
EFW: IV Fluid volume − ((Na^+^ mmol + K^+^ mmol)/140)
EFW: Fluid volume − ((Na^+^ mmol + K^+^ mmol)/140)
This accounts for both the infused and excreted volume. The Na+ and K+ concentrations correspond to the respective volumes
**Substudy B**
*GRIP Potassium Intake = *GRIP prescribed potassium chloride in mmol
*Potassium Output = *RKE = 24 h potassium excretion in the urine in mmol
*GRIP Potassium balance = *GRIP potassium intake − potassium output
**Substudy C**
*Fluid intake* = infusion fluids + given medication + water (oral)
*Fluid output = *gastric retention + drain production + diuresis (24 h urines)
*Balance* = fluid intake − fluid output

**Table 2:** Electrolyte content of infusion fluids used in substudy A

	[K^+^] (mmol/L)	[Cl^−^] (mmol/L)	[Na^+^] (mmol/L)
*Resuscitation fluids*			
Voluven^®^	0	154	154
Sterofundin^®^	4.02	127	145
Lactated Ringers	5.4	111	134
NaCl 5%	0	856	856
Glucose 5%	0	0	0
Glucose 50%	0	0	0
Glucose 2.5%/NaCl 0.45%	0	77	77
NaCl 0.9%	0	154	154
*Parenteral/enteral feeding*			
Nutrison protein plus^®^	42.97	22.57	48.26
Nutrison concentrated^®^	49.86	22.57	43.5
Nutrison multifibre^®^	38.36	35.27	43.5
Nutridrink^®^	39.15	40.67	24.54
Peptisorb^®^	38.4	35.27	43.5
TPN	30	45	35
*Blood products*			
RBC	40	80	126
FFP	2	80	172
Thrombocyte concentrate	2	70	120
Cirrestor blood	4	0	140
Cell saver blood	0	100	140
Albumin 20%	0	100	100
Fibrinogen	0	0	71
Thrombocyte concentrate	2	70	120

**Table 3:** Solutions used to dissolve frequently used medication in substudy A

Type of medication	Dissolved in infusion fluid^a^
Propofol 2%	None
Midazolam 100 mg/50 mL	NaCl 0.9%
Morphine 100 mg/50 mL	NaCl 0.9%
Insulin 50 IU/50 mL	NaCl 0.9%
Noradrenaline 10 mg/50 mL	Glucose 5%
Adrenaline 10 mg/50 mL	NaCl 0.9%
Dobutamine 250 mg/50 mL	NaCl 0.9%
Dopamine 200 mg/50 mL	NaCl 0.9%
Amiodarone 600 mg/50 mL	Glucose 5%
Nicardipin 10 mg/50 mL	NaCl 0.9%
Milrinone 10 mg/50 mL	NaCl 0.9%
Magnesium sulfate	NaCl 0.9%
Furosemide 80 mg/50 mL	NaCl 0.9%
Nitroglycerin 10 mg/50 mL	NaCl 0.9%
Vasopressin 40 U/40 mL	NaCl 0.9%
Tacrolimus 2 mg/50 mL	NaCl 0.9%
Sodium phosphate	NaCl 0.9%
Dexmedetomidine	Glucose 5%
Clonidine 600 *μ*g/50 mL	NaCl 0.9%
Hydrocortisone 200 mg/50 mL	NaCl 0.9%
Heparin 20,000 IU/50 mL	NaCl 0.9%
Piperacillin/Tazobactam (4/500)	Water ([Na^+^]_end_ = 196 mmol/L)
Flucloxacillin	NaCl 0.9% ([Na^+^]_end_ = 418 mmol/L)
Naloxone	NaCl 0.9%
Tranexaminic acid	NaCl 0.9%
Labetalol 250 mg/50 mL	None
Mycophenolate mofetil	Glucose 5%
Ganciclovir	NaCl 0.9%
Levosimendan	Glucose 5%
Protamine	NaCl 0.9%
Phenylephrine	NaCl 0.9%

a Infusion fluids according to our institutions protocol.

*Substudy B* evaluated patients who stayed ≥24 h at the ICU after cardiac surgery and who were targeted to a serum potassium of either 4.0 or 4.5 mmol/L using our GRIP‐II protocol. Patients targeted at 4.0 or 4.5 mmol/L were compared after selection and matching for admission reason, disease severity and length of ICU‐stay. The differences between the cumulative GRIP‐II prescribed potassium chloride dose and cumulative 24 h renal potassium excretion (RKE) were compared between the two target groups (Table 1).

*Substudy C* was a predefined analysis of the GRIP‐COMPASS trial (Hoekstra et al. 2010, 2016) in patients with an ICU‐stay of >4 days. GRIP‐COMPASS assessed the impact of the 4.0 and 4.5 potassium targets on the incidence of atrial fibrillation. Here, we analyzed the effect of the 4.0 or the 4.5 mmol/L targets on fluid balances as calculated from intake of IV fluids, nutrition, and medication, and losses by diuresis, gastric retention, and drain production (Table 1).

### Data collection

Analyzed data included basic demographics, reason of admission, acute physiology, and chronic health evaluation (APACHE‐IV) score for disease severity, acute kidney injury according to the KDIGO AKI criteria (Kellum and Lameire 2013) and in‐hospital mortality. All electrolyte, glucose and pH values, determined in blood or 24 h urine during the first four ICU days were recorded. Samples that displayed hemolysis or otherwise were deemed less reliable, were excluded from analysis.

### Balance calculations

Fluid and electrolyte balances were derived from patient charts taking the electrolyte content of administered fluids, medication, and nutrition into account (Tables 1, 2, 3). Insensible perspiration was defined as loss through the skin by evaporation and evaporative water loss from the respiratory tract (Cox 1987). We did not take losses via sweat and stool into account.

We corrected for intubation, since loss of fluid will be lower when intubated. Since the admission day is typically not a full day in most cases, this was corrected for in the calculated insensible perspiration.

Electrolyte‐free water was determined for both the administered and the lost or excreted volumes by taking the total infused or excreted volume and subtracting the total amount of Na^+^ and K^+^ infused or excreted (Rose 2001; Lindner et al. 2009). This accounts for both the administered and excreted volume. Na^+^ and K^+^ concentrations of 140 mmol/L were used to determine corresponding electrolyte containing volumes. EFW was estimated only on the basis of the cations Na^+^ and K^+^. Other cations (e.g., Ca^++^ and Mg^++^) were not taken into consideration since these cations form only a minor fraction of administred fluids. Also, ICV and ECV contain only minor amounts of these cations in a readily exchangeable form.

### Statistical analysis

Means are given ±SE, unless indicated otherwise, medians with interquartile range. Baseline characteristics between groups were compared using a chi‐square or a Mann–Whitney *U*‐test. Balances and electrolyte levels were compared with the Student's *t*‐test. A two‐sided *P* < 0.05 was considered significant. Balance calculations were performed with a spreadsheet (Excel, Microsoft, Redmond, WA) and statistical analyses were performed with SPSS 22 (IBM, Chicago, IL).

### Study approval

The data analysis in this study was performed in accordance with the guidelines as outlined in Dutch legislation. The study was approved by the medical ethics committee (IRB) of our institution (Medisch Ethische Toetsingcommissie, METc 2015.089). As a retrospective study of routinely collected and anonymized data, informed consent was not required by our IRB. The GRIP‐COMPASS trial is registered at Clinicaltrials.gov (NCT01085071).

## Results

### Substudy A: comprehensive balance analysis

**Figure 1:**
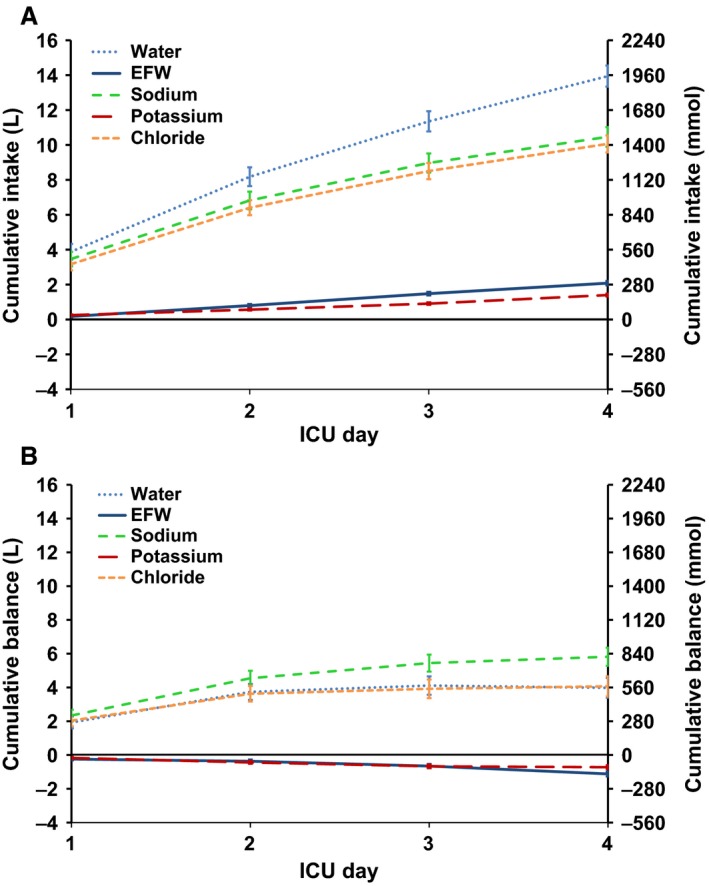
Cumulative fluid and electrolyte intake and balances in 39 patients in substudy A over the first 4 ICU days. All panels depict the mean ± SE for the first 4 ICU days. The 2 L and 280 mmol multiples on two Y‐axes were chosen to match the normal [Na^+^] of 140 mmol per 1 L, in order to reflect the associated volumes of the intracellular volumes (ICV) and extracellular volumes (ECV). (A) Cumulative intakes show that patients received considerable amounts of fluid, sodium, and chloride as well as potassium and electrolyte‐free water (EFW). (B) Cumulative balances show that fluid, sodium, and chloride were retained, but no retention of EFW and potassium occurred. This indicates that ICV remains constant or even shrinks, while the ECV is expanding.

Cumulative intake and balances were collected of 39 ICU patients (Table 4) for a 4‐day period. Over this period, large amounts of fluid (14.0 ± 0.6 L), EFW (2.0 ± 0.1 L), sodium (1465 ± 79 mmol), potassium (196 ± 11 mmol), and chloride (1408 ± 69 mmol) were administered (Fig. 1A). A positive cumulative fluid balance of +4.0 ± 0.6 L was seen with positive sodium and chloride balances of +814 ± 75 and +569 ± 83 mmol, respectively. In contrast, there was a net potassium balance of −101 ± 14 mmol and a net EFW balance of −1.1 ± 0.2 L (Fig. 1B).

**Table 4:** Patient characteristics of substudies A, B, and C*

	Substudy A	Substudy B			Substudy C		
	(*n* = 39)	4.0 (*n* = 229)	4.5 (*n* = 297)	*P*	4.0 (*n* = 54)	4.5 (*n* = 63)	*P*
Age, year, mean (SD)	65 (15)	67 (12)	67 (13)	0.52	68 (11)	63 (17)	0.30
Sex, male	29 (74%)	149 (65%)	210 (71%)	0.17	25 (46%)	42 (67%)	0.03
Reason of admission							
Cardiothoracic surgery	32 (82%)	211 (95%)	263 (89%)	0.19	42 (78%)	54 (86%)	0.27
Trauma	1 (3%)	3 (1%)	2 (1%)		0 (0%)	(0%)	
Vascular surgery	1 (3%)	0 (0%)	0 (0%)		0 (0%)	(0%)	
Miscellaneous	5 (13%)	15 (7%)	32 (11%)		12 (22%)	9 (14%)	
LOS ICU, d, median (IQR)	7.0 (4.0–13.1)	4.7 (2.8–8.0)	4.7 (3.0–8.9)	0.28	10.0 (5.7–19.9)	9.8 (4.9–15.6)	0.41
APACHE‐IV, median (IQR)	61 (45–72)†	58 (47–67)	59 (45–71)^‡^	0.56	57 (49–69)	52 (42–65)¶	0.07
Hospital mortality	4 (10%)	22 (10%)	28 (9%)	0.95	12 (22%)	10 (16%)	0.38
AKI	11 (28%)	78 (36%)	82 (32%)§	0.44	12 (26%)	26 (45%)¶	0.04
Stage 1	6 (55%)	45 (58%)	49 (60%)		6 (50%)	16 (36%)	
Stage 2	3 (27%)	19 (24%)	16 (20%)		6 (50%)	9 (20%)	
Stage 3	2 (18%)	14 (18%)	17 (21%)		0 (0%)	1 (2%)	
Diuretic use	25 (64%)	–	–		18 (33%)	26 (41%)	0.38
pH, median (IQR)	7.40 (7.37–7.41)	–	–		–	–	
Glucose, mmol/L, median (IQR)	7.7 (7.4–7.9)	–	–		–	–	

*AKI, acute kidney injury; APACHE‐IV, acute physiology and chronic health evaluation‐IV; LOS, length of stay; ICU, intensive care unit; IQR, interquartile range; ^†^for 33 (85%) patients; ^‡^for 486 (92%) patients; ^§^for 471 (90%) patients; ^¶^for 105 (90%) patients.

**Figure 2:**
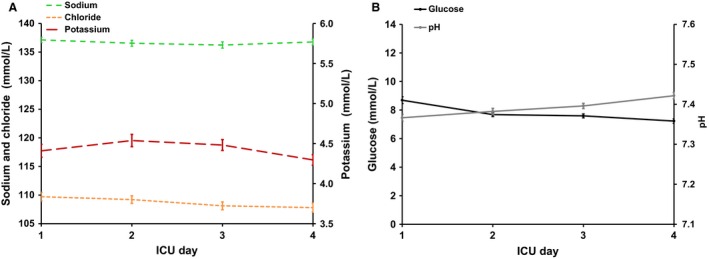
Circulating electrolyte, glucose, and pH levels in substudy A. (A) Mean ± SE circulating concentrations of sodium (reference range 135–145 mmol/L), potassium (3.5–5.0 mmol/L) and chloride (97–107 mmol/L) are shown. During the first 4 ICU days, electrolyte concentrations were stable (Kruskal–Wallis test; *P*= NS). (B) Mean ± SE circulating concentrations of glucose (reference range 4.0–6.4 mmol/L) and pH (7.35–7.45 mol/L) are shown. During the first 4 ICU days, glucose levels decreased, while pH showed a little rise (both glucose and pH; Kruskal–Wallis test; *P* < 0.001).

Blood electrolyte concentrations were stable during the study period (Fig. 2A). Glucose levels were mildly hyperglycemic with a decrease of 1.5 mmol over the first 4 ICU days (Fig. 2B). Arterial pH levels stayed within the reference range (Fig. 2B).

### Substudy B: effect of two different potassium targets on potassium balance

**Figure 3:**
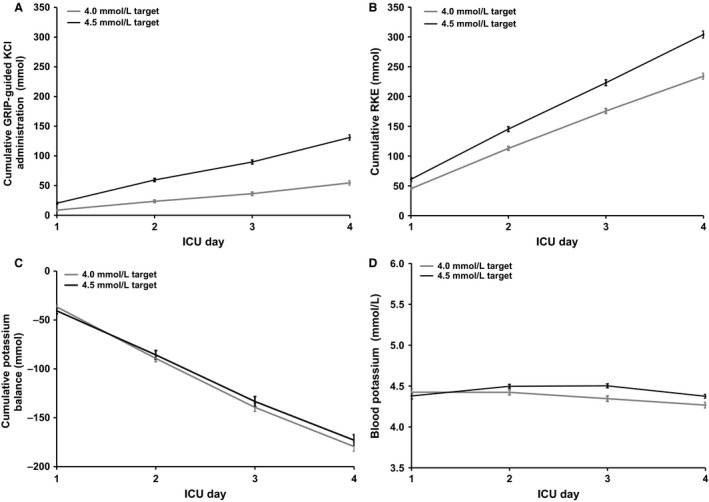
Potassium infusion, excretion, balances, and blood potassium in substudy B for both the 4.0 (*n* = 920) and 4.5 mmol/L target groups (*n* = 1162). All panels depict the mean ± SE for the first 4 ICU days. (A) Cumulative potassium infusion with the 4.5 mmol/L target group receiving 76 mmol (42%) more potassium than the 4.0 group (Student's *t*‐test; *P* < 0.001). (B) Cumulative potassium excretion, with the 4.5 mmol/L target group excreting 70 mmol more (Student's *t*‐test; *P* < 0.001). (C) Cumulative potassium balances are progressively negative. The similarity of the two target groups indicates shows that the additionally infused potassium is not retained (Student's *t*‐test; *P* = 0.42). (D) Blood potassium was only slightly higher in the 4.5 mmol/L target group despite a 42% higher potassium administration in this group compared to the 4.0 mmol/L target group. The mean blood potassium concentration only differed 0.07 mmol between the two groups (Student's *t*‐test; *P* < 0.001).

GRIP‐prescribed potassium infusion, RKE, and potassium balances were determined for 526 cardiothoracic ICU patients (229 patients targeted at the 4.0 mmol/L potassium target and 297 patients targeted at 4.5 mmol/L potassium target) with no baseline differences (Table 4). The cumulative infused potassium dose was 76 ± 23 mmol higher (Fig. 3A) and the RKE was 70 ± 23 mmol higher in the 4.5 mmol/L target group compared to the 4.0 mmol/L target group (Fig. 3B).

Both groups showed similar negative potassium balances (*P* = 0.42, Fig. 3C). Furthermore, blood potassium levels only showed a slight difference between both groups (*P* < 0.001; Fig. 3D).

### Substudy C: effect of two different potassium targets on fluid balance

**Figure 4:**
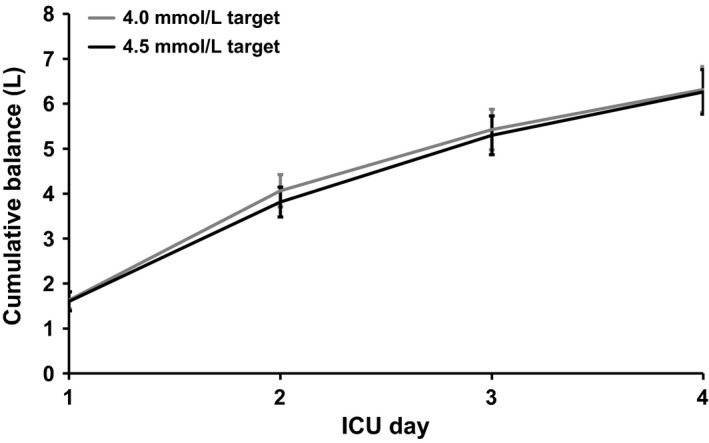
Cumulative fluid balances in patients in substudy C. Mean ± SE cumulative fluid balances (i.e., net fluid received) for the first 4 ICU days for both the 4.0 (*n* = 54) and 4.5 (*n* = 63) mmol/L target group are shown. Despite a higher potassium administration rate in the 4.5‐group, the strongly positive fluid balances did not differ between the 4.0 and 4.5 mmol/L target groups (Student's *t*‐test; *P* = 0.61)

Fluid balances in 117 patients (54 patients targeted at the 4.0 mmol/L potassium target and 63 patients targeted at the 4.5 mmol/L potassium target) were examined. The patient groups had similar baseline characteristics (Table 4) and were admitted for at least 5 ICU days with a median of 10 ICU days. Net fluid balances after four ICU days did not differ between the two groups (6.3 ± 0.4 and 6.3 ± 0.4 L, respectively; *P* = 0.61) despite receiving significantly different amounts of potassium (Fig. 4).

## Discussion

In this first study using comprehensive balances to examine the conventional model on the distribution of fluid and electrolytes over the ECV and ICV, we found a rapid and profound volume expansion of the ECV, while the ICV did not expand.

In substudy A, we observed a large cumulative positive balance of fluids, sodium, and chloride, whereas there was a negative balance of both potassium and EFW. This indicates that no increase of the ICV occurred, since such an increase in ICV should have been accompanied by intracellular potassium retention and thus a positive potassium balance. Additionally, blood electrolytes remained stable during this period. Since intra‐ and extracellular osmolality are essentially equal, this corroborates that no increase in ICV occurred. Both the renal excretion of all the additionally administered potassium in patients targeted at 4.5 mmol/L in substudy B, as well as the absence of more positive fluid balances in patients targeted at 4.5 mmol/L in substudy C underscore that the ICV is not affected by additionally administered potassium. That the extra administered potassium is not retained but excreted, also explains the similarity in potassium levels that was observed in the prospective GRIP‐COMPASS trial (4.22 ± 0.36 vs. 4.33 ± 0.36; *P* < 0.001) (Hoekstra et al. 2010, 2016). In fact, the overall negative potassium balance implies a decrease in TBK and thus a contraction of the ICV. This has been observed in trauma patients (Finn et al. 1996). A major contributor to the loss of ICV and thus potassium is the rapid breakdown of striated muscle tissue that is frequently observed in catabolic critically ill patients (Finn et al. 1996; Monk et al. 1996; Savalle et al. 2012).

A perfect quantitative measurement for the ICV does not exist. However, determination of TBK is still considered the best measurement of ICV (Patrick 1977; Finn et al. 1996; Monk et al. 1996; Dittmar and Reber 2004; Savalle et al. 2012). The current gold standard to assess TBK is scintigraphy of ^40^K exploiting the fact that all naturally occurring potassium contains a minute and constant fraction of ^40^K, a radioactive isotope, allowing the determination of TBK with an accuracy in the order of several percent (approximately 100 mmol) (Samat et al. 1997). NaBr is sometimes used together with 40K to determine the ICV as well as body composition (Shen et al. 2005; Savalle et al. 2012), but this method requires a stable body water pool size rendering it unsuitable in ICU patients. A more popular and less cumbersome, but very indirect and considerably less reliable method to estimate ICV and body composition is bio‐impedance analysis (BIA) (Dittmar and Reber 2004; Savalle et al. 2012). BIA is difficult to interpret in ICU patients and is particularly poorly suited to detect small changes in ICV. BIA tends to overestimate body cell mass in comparison to TBK by up to 20% and BIA devices have several systematic errors (Dittmar and Reber 2004). We are not the first to propose potassium balances as an easy and reliable way to measure changes in TBK (Patrick 1977; Finn et al. 1996; Monk et al. 1996). But to our knowledge, we are the first to propose potassium balances as a direct measure of changes in ICV in patients who undergo dramatic volume and electrolyte shifts. The measurement of RKE, essential for calculating the potassium balance, is widely available, inexpensive and noninvasive in ICU patients who typically already possess a urinary catheter, which would make this method more feasible for current practice than previously described methods. Thus, whereas ^40^K scintigraphy is most accurate in measuring absolute TBK, the potassium balance method may be optimal to determine changes in TBK and therefore may also be serve as an indicator of muscle loss in ICU patients.

An important clinical implication from our observations concerns the strong preference within clinical medicine for sodium‐based intravenous fluids over EFW solutions, such as glucose 5%, as the former are considered to expand ECV without significant expansion of ICV as compared to EFW solutions (Rose 2001; Guyton 2011; Frost 2015). Large infusions of sodium‐based fluids frequently lead to sodium accumulation and hypernatremia in patients. Hypernatremia in the ICU is thus largely iatrogenic and it has a strong correlation with negative outcomes (Gosling 2003; Stelfox et al. 2008; Lindner et al. 2009; Silversides et al. 2010; Oude Lansink‐Hartgring et al. 2016). In this study we found no indicators of ICV expansion following administration of EFW. Consequently the need for so‐called “physiological” sodium‐based infusion fluids (i.e., 130–154 mmol/L) can be called into question. However, this does require further investigation since our study was not designed to directly compare different fluid regimens (e.g., sodium‐free solutions, low‐chloride solutions). If this is also applicable to patients outside of the ICU who receive iv infusions, cannot be concluded yet.

**Figure 5:**
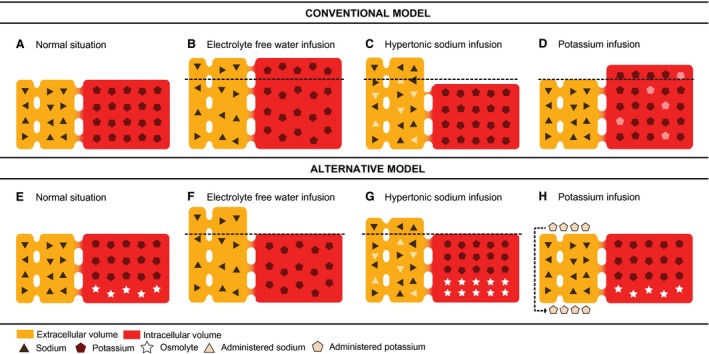
Conventional and alternative simplified models on water, sodium, and potassium distribution. Note that both models do not include muscle loss, causing a ‘structural decrease’ of the ICV. Under either model water, potassium, and sodium are freely exchanged between the extracellular volume (ECV; yellow: plasma and interstitium) and intracellular volume (ICV; red), governed by physico‐chemical principles. (A through D) Conventional model depicting the normal distribution of sodium (triangles) and potassium (pentagons). (B) Water distribution after the administration of electrolyte‐free water (EFW; e.g., glucose 5% infusion). The additional water is proportionally distributed over the ECV and ICV. (C) Administration of a hypertonic sodium infusion, which homes to the ECV. The osmotic equilibrium is achieved by redistribution of water from the ICV to the ECV. (D) Administration of an isotonic potassium infusion, that in case the potassium is retained by the body, should home to the ICV. As this is a simplified model, the additional response to the potassium infusion namely the extrusion of sodium is left out. (E through H): Alternative model that incorporates intracellular osmolytes (stars), which are osmotically active solutes that are dynamically generated or cleared by the cell. The ICV is kept constant by varying the intracellular osmolyte content to match extracellular osmolarity. (F) Water distribution after administration of EFW. Note that the ICV has cleared its osmolytes to keep its volume constant and maintain iso‐osmolarity with the ECV. (G) A hypertonic sodium infusion stays in the ECV. The ICV generates osmolytes to keep its volume constant and increase its osmotic pressure to the same level as the ECV. (H) An isotonic potassium infusion does not enter the ICV and additional potassium is thus renally excreted.

Although major textbooks on physiology (Guyton 2011) and electrolyte and water pathophysiology (Rose 2001) and a recent review (Frost 2015) claim that EFW distributes proportionally over the ICV and ECV (Fig. 5A–D), this concept has not been verified in critically ill patients who require extensive IV fluid administration in the context of a systemic inflammatory response. This conventional model has its origins in ex vivo erythrocyte experiments, first executed by William Hewson in 1773 (Kleinzeller 1996). Hewson's observations that erythrocytes swell in water and shrink in a hypertonic solution would later lead to recognition of osmotic pressure as a key determinant of cellular volume. Although very important from a mechanistic point‐of‐view, these in vitro experiments where cells are abruptly exposed to extremely hypo‐ or hyperosmolar solutions cannot be extrapolated to changes in vivo, where cells are more gradually exposed to less extreme osmotic stress.

Maintaining a constant volume, however, is critical for cellular homeostasis since volume changes affect many critical metabolic and signaling processes (Yancey et al. 1982; Chamberlin and Strange 1989; Strange 2004). Most life forms, from bacteria to eukaryotes, have developed evolutionarily highly conserved mechanisms to rapidly adjust the concentration of so‐called osmolytes (Yancey et al. 1982; Chamberlin and Strange 1989; Lang et al. 1998; Strange 2004; Lang 2006; Hoffmann et al. 2009). Osmolytes are comparatively inert intracellular molecules including sugars, polyols, amino acids, urea, and methylamines, that can be generated and removed on short notice to avert shrinking and swelling in hyper‐osmolar or hypo‐osmolar environments. The initial responses on changing extracellular environments are regulatory volume decrease or regulatory volume increase, in which the cell is forced to release or gain potassium, which triggers the generation or clearance of nonessential osmolytes, in order to restore the cell volume (Strange 2004; Hoffmann et al. 2009). Figure 5E–H shows an alternative model that is both compatible with extensive evidence from cell biology on the role of osmolytes and our findings in vivo. The key difference of the alternative model compared to the conventional model is the relative constancy of the ICV. The “*milieu interieur*” that animals possess (i.e., the ECV) varies its volume and osmolarity while cells maintain constant volume by adapting the osmolyte concentration. Note that both simplified models shown in Figure 5, do not take structural loss of striated muscle tissue and consequently diminished ICV into account (Finn et al. 1996; Monk et al. 1996).

Our study has several limitations. As a retrospective study, many variations in standard care could not be controlled for. Since we did not possess a patient database management system during the study, very time‐consuming calculations of balances from nonelectronic patient charts had to be performed. The later introduction of routine 24 h urine analysis led us to split our study into three complementary substudies to obtain the relevant data. On the other hand, ICUs that regularly perform the inexpensive yet accurate 24 h urine analyses and that do possess a patient database management system could automatically determine the relevant balances in nearly real time. Although we meticulously determined the electrolyte and fluid balances in substudy A, we had to make several assumptions such as those regarding insensible water losses. However, given the consistent and marked results, we conclude that errors introduced by these assumptions will only slightly affect the overall differences or the absence of differences in the observed balances. We did not account for fecal and other potassium losses. Inclusion of these unmeasured losses would have resulted in even more profound potassium losses, indicative of an even larger decrease in ICV. Significant changes in glucose and pH are known to alter the distribution of potassium (Aronson and Giebisch 2011; Palmer 2015). As described, pH was stable and mostly in the normal range, glucose was only mildly increased. We therefore believe that these factors are unlikely to have affected potassium distribution. Data on the perioperative phase would have been very interesting, but balance information during surgery was incomplete, thus we could only assess the postoperative phase. It would be interesting to elucidate the counterregulatory mechanisms that interfered with actually achieving the 4.0 and 4.5 targets, including factors that control RKE in response to higher potassium loads or pharmacological interventions. But this was neither the goal nor feasible in this study. With respect to this issue, it should be stressed that a key methodological advantage of balance studies is that they do not require any specific assumption on the obviously complex underlying homeostatic systems. Prospective studies in patients who do not have such large fluid requirements and who do not display such pronounced loss of muscle mass as our patients would be welcome. In such patients balance studies could compare the effects of electrolyte‐based fluids (e.g., NaCl 0.9%) with more EFW‐based fluids (e.g., NaCl 0.45%/glucose 2.5%).

In conclusion, in this first study to comprehensively examine fluid and electrolyte balances in patients during marked volume expansion after ICU admission, we could not demonstrate retention of administered EFW and potassium. Moreover, significant potassium losses were observed, indicating ICV contraction. On the other hand, administered sodium and accompanying fluids were retained, indicating concomitant ECV expansion.

## Conflict of Interest

The authors have declared that no conflict of interest exists.
